# Public Health Infection Prevention Mentorship Program for Philadelphia Skilled Nursing Facilities Utilizing a Health Equity Framework

**DOI:** 10.1017/ash.2025.201

**Published:** 2025-09-24

**Authors:** Tiina Peritz, Nicole Brand, Jessica M Rice, Jenna Scully, Kathryn Hitchcock, DeAnn Richards, Jane M Gould

**Affiliations:** 1 Philadelphia Department of Public Health (PDPH) Healthcare-Associated Infections and Antimicrobial Resistance (HAI/AR) Program, Philadelphia, PA, USA; 2 APIC Consulting, Inc., Arlington, VA, USA

## Abstract

**Background:** Skilled nursing facilities (SNFs) face many challenges implementing robust infection prevention and control (IPC) programs. The Philadelphia Department of Public Health (PDPH) partnered with APIC Consulting Services, a wholly owned subsidiary of The Association for Professionals in Infection Control and Epidemiology (APIC), to provide IPC mentoring to Philadelphia SNFs. The objective of the program was to strengthen IPC capacities by providing an in-depth IPC assessment followed by an action plan and longitudinal infection preventionist (IP) support to mitigate identified gaps. **Methods:** A health equity framework based on area deprivation index (ADI), percent of residents on Medicaid, and Centers for Medicare & Medicaid (CMS) star rating was developed to identify priority SNFs for recruitment into the voluntary program. Participating SNFs received a three-day onsite IPC assessment with an expert IP consultant using an expanded version of the Centers for Disease Control Infection Control Assessment and Response (ICAR) tool. Assigned consultants provided mentorship and education for the SNF IP for up to six months. Each facility identified 4-5 focus areas and co-developed an action plan with the consultant. SNF assessment data collected July 2023 -May 2024 were analyzed to assess IPC gaps across facilities. **Results:** Participants included 11/46 (24%) Philadelphia SNFs, including 8/18 (44%) priority facilities. Median facility size was 189 beds and median census was 164 residents. Program completion rate was 73%. Consultants performed 66 onsite visits and 26 remote visits, totaling over 1,752 hours of support. Median number of IPC gaps identified was 79 (IQR: 57-84), most frequently within the domains of environmental cleaning and disinfection (13%); water management (10%); and training, auditing, and feedback (9%). Common facility-chosen action plan focus areas included disease surveillance (24%), antibiotic stewardship (16%), and hand hygiene (13%). Main barriers to program completion included lack of leadership support (18%) and staff turnover (9%). **Conclusions:** Expert-driven longitudinal support can be an effective strategy for enhancing IPC capacity within low resourced SNFs and a data-based health equity framework can be used to prioritize facilities for support. Through targeted mentorship, this program identified and addressed gaps in IPC practices and fostered a culture of safety. Most common action plan focus areas selected by the facilities did not align with IPC topic areas where most recommendations were given, highlighting potential SNF IPC program areas that may be challenging for facilities to address and where further education and resources are needed.

